# Passive Fit Evaluation of Implant Superstructures by Analyzing Accumulated Screw Tightening Torque: A Dental Technique

**DOI:** 10.1155/crid/9268381

**Published:** 2025-10-29

**Authors:** Kazuya Doi, Hiroshi Oue, Reiko Kobatake, Kaien Wakamatsu, Kazuhiro Tsuga

**Affiliations:** Department of Advanced Prosthodontics, Graduate School of Biomedical and Health Sciences, Hiroshima University, Hiroshima, Japan

**Keywords:** accumulated torque, case report, passive fit, screw tightening torque

## Abstract

Achieving a stable passive fit is important in implant treatment, especially for restorations of screw-retained superstructures. A passive fit is often evaluated by tactile sensing of tightening a fixing screw; however, tactile sensing is a subjective procedure. Objective and quantitative assessment of passive fits can be performed by measuring the tightening torque on prosthetic screws. In this clinical case, accumulative screw tightening torques for passive fit and misfit superstructures were calculated to examine the usefulness of the proposed method. The results suggested that accumulative torques and time slopes of torques may be potentially useful parameters for quantitatively evaluating passive fits; further laboratory and/or clinical trials are necessary.

## 1. Introduction

For the successful long-term prognosis of implant treatment, the fit between the implant body and the prosthetic component is important. Achieving a passive fit is particularly important when multiple prostheses are screwed onto an implant body [1]. Passive fit refers to a state when the implant body and prosthetic components are connected without mechanical stress. If a passive fit with screws retaining multiple prosthetic components cannot be achieved, distortion of prosthetic components may occur owing to stress during screw tightening. The distortion of misfit prosthetic components places a continuous load on the implant body, increasing the risk of osseointegration loss [2]. Furthermore, misfit of implant-supported restorations can result in gaps around the implants, leading to a risk of peri-implantitis via microleakage [3].

The passive fit between an implant body and prosthetic component is evaluated using intraoral radiographs and interpretation of tactile sensation while tightening the retaining screws. Passive fit evaluation using tactile sensation of screw tightening is a simple and direct evaluation method that is widely used in clinical practice [4]. However, tactile sensation is a subjective approach because it depends on personal perception and experience of an operator [5]. Furthermore, evaluation of a passive fit based on tactile sensation cannot be objectively quantified. Therefore, the precision of fit between a superstructure and implants is difficult to assess clinically. On the other hand, torque-controlled surgical motors are being utilized for performing a quantitative assessment of passive fits [6, 7].

Figueras-Alvarez et al. reported that a passive fit can be evaluated by measuring the prosthetic screw tightening torque and analyzing the torque/time graph obtained during the placement of the retaining screws with a torque-controlled surgical motor, which can be used as an effective quantity for objective evaluation [8]. Our previous study demonstrated that the tightening torque can be accurately measured even at low bone-density sites using a torque-controlled surgical motor operated using a computer-based program [9]. The tightening torque method enables the detection of low torque values even at short time intervals. This study reports the first clinical case of a passive fit evaluation performed by measuring the accumulative torque on retaining screws and determining the presence of misfit of screw-retained superstructures.

## 2. Case Presentation

### 2.1. Case

**Figure 1:**
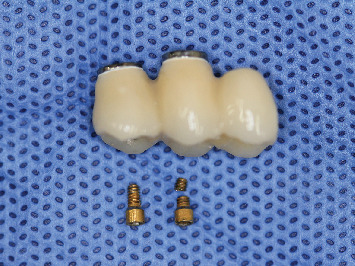
The removed monolithic zirconia–based superstructure on a titanium base and prosthetic gold screws. The fractured screw is the mesial screw.

Written informed consent was obtained from the patient to publish this report in accordance with the journal's patient consent policy. A 71-year-old woman who underwent implant treatment was the subject for passive fit evaluation. The main complaint of the subject was regarding the mobility of the implant superstructure, which was reported to be unstable immediately after the implant treatment. The patient had no notable medical, family, or psychosocial history. Intraoral findings demonstrated two implants in the maxillary right molar area and a cantilever-type screw-retained zirconia superstructure set on the abutment level. The superstructure was removed for condition assessment. Inspection of the superstructure showed a fracture in the proximal prosthetic gold screw (Figure 1). The superstructure was then secured to the abutment level using a new prosthetic titanium alloy screw (Prosthetic Screw Multi-unit, Nobel Biocare, Kloten, Switzerland). The x-ray assessment of the fit conditions after fitting the superstructure demonstrated a misfit between the abutment level of the implant body and superstructure. Therefore, a provisional resin-based superstructure was fabricated using the open-tray technique by connecting polymethyl methacrylate to a temporary cylinder (Temporary Coping Multi-unit, Nobel Biocare). To compare the fit conditions of the old and new superstructures, intraoral radiographs were recorded. Furthermore, the tightening torques exerted on the prosthetic screws using a working model were measured and compared.

### 2.2. Methods

#### 2.2.1. Fit Evaluation of Newly Fabricated Provisional Superstructure

The newly fabricated provisional resin-based superstructure was fixed to the working cast model using hand-tightened screws. Subsequently, the distal screw was loosened by one turn. The surgical motor (Surgic Pro2, Nakanishi Inc., Tochigi, Japan) was then used to automatically tighten the screw at a maximum setting of 15 Ncm and 10 rpm to measure the tightening torque. Next, the mesial screw was loosened by one turn and the automatic tightening torque was measured under the same conditions. During automatic tightening, the torque was recorded in intervals of 0.05 s. The contact between the buccal surface of the superstructure and working cast model was photographed using a digital camera (EOS Kiss X4, Canon Inc., Tokyo, Japan) positioned parallel to the subject to allow visualization of the gap between the implant and the superstructure. Subsequently, the new provisional superstructure was delivered to the patient, and a radiograph was taken using the paralleling technique. This was compared with the radiograph of the previously fabricated misfit superstructure.

#### 2.2.2. Fit Evaluation of Previously Fabricated Superstructure

The misfit superstructure was fixed to the working cast model using hand-tightened screws. Initially, the quality of the passive fit was not assessed. Thus, using the same technique as that described in Section 2.2.1, the automatic tightening torques exerted on the proximal and distal screws were measured. The contact between the buccal surface of the superstructure and the working cast model was photographed using a digital camera.

### 2.3. Results

#### 2.3.1. Comparison of Fit Conditions Based on Intraoral Radiographs and Working Cast Model

**Figure 2:**
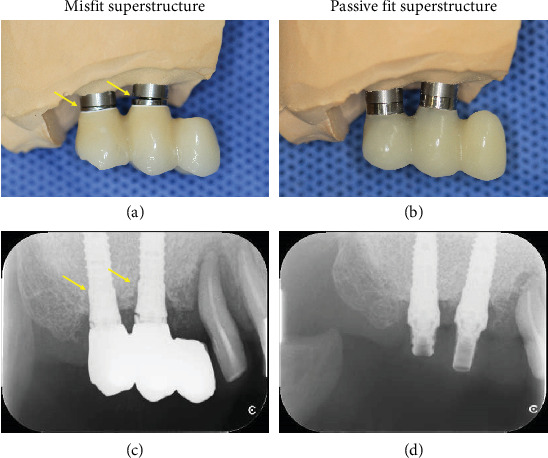
Fit evaluation of the superstructure using an intraoral working cast model and radiograph analysis (yellow arrows indicate micro gap). (a) Image showing the misfits between the previously fabricated superstructure and the abutment analog. (b) Image showing the fit between the resin-based provisional superstructure and the abutment analog. (c) Radiograph showing misfits of the previously fabricated superstructure and the abutment level of the subject. (d) Radiograph showing an almost perfect fit between the fabricated provisional superstructure and the abutment level of the subject.

In the working cast model, a gap can be observed between the implant abutment analog and the previously fabricated superstructure (Figure 2a), whereas no gap can be observed between the implant abutment analog and the newly fabricated superstructure (Figure 2b).

Similarly, intraoral radiograph findings demonstrate a gap between the abutment level of the subject and the previously fabricated superstructure (Figure 2c), whereas no gap can be observed between the abutment level of the subject and the newly fabricated superstructure (Figure 2d).

#### 2.3.2. Comparison of Fit Conditions Based on Accumulated Tightening Torques

**Figure 3:**
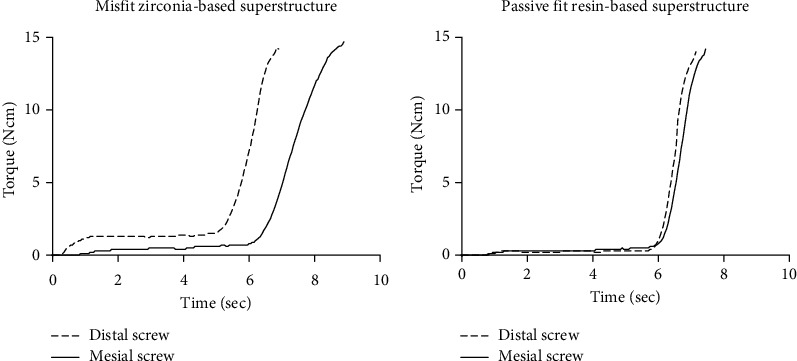
Overlay graph of screw tightening torques as a function of time after placing the retaining misfit and passive fit superstructures. (a) Misfit zirconia-based superstructure. (b) Passive fit resin-based superstructure.

The torques corresponding to each prosthetic screw, measured at intervals of 0.05 s, were plotted on a graph to show the waveform. Additionally, the torque accumulated up to the maximum torque limit (15 Ncm) calculated and listed in Table 1. In the misfit superstructure, the screw tightening torque on each side showed a different slope (Figure 3a). The slope of the torque versus time graphs obtained on the misfit superstructure can be observed to gradually reach the maximum values. Compared to the torque observed during distal tightening of the misfit superstructure, the torque observed during mesial tightening can be observed to reach its maximum value more slowly, indicating a misfit alignment.

On the contrary, fit superstructure, the screw tightening torque on each side showed a similar slope (Figure 3b). The slope of the torque versus time graphs obtained on the fit superstructure can be observed to steeply reach the maximum values. The waveforms of the graphs presented in passive fit are nearly identical for the distal and mesial screws. In terms of the accumulative torques, the misfit superstructure exhibits larger values than those exhibited by the passive fit superstructure.

## 3. Discussion

Passive fit assessment was performed using parallel imaging to confirm the presence of gaps between the superstructure and implant body or abutment level. Parallel imaging is useful for evaluating areas that cannot be viewed directly, such as subgingival areas; however, evaluating minute gaps is difficult and the evaluation may be inaccurate depending on the shooting angle [10]. Assessing screw tightness is a direct passive fit evaluation method that is widely used in clinical practice. Several methods have been designed and demonstrated to evaluate passive fit by assessing screw tightness, including visual assessment using the 1-screw or Sheffield fit tests [4]. When evaluating the sensation of tightness, relatively large loads can be felt in areas where the joint between the superstructure and implant body is incompatible. The fit evaluations performed based on the sensation of tightness are subjective. However, the fit evaluations performed using torque measurements allow for objective and quantitative evaluations. Passive fit assessments using screw tightening torque measurements require detecting relatively small values of torques and examining the variations in torque over time.

Figueras-Alvarez et al. have introduced a method for measuring torque using a torque-controlled surgical motor to evaluate passive fit [8]. This report mentions the possibility of evaluating passive fit based on the correlation between torque value and rotation speed; however, the torque value was not measured at detailed intervals to evaluate the fit. Detailed screw tightening torque measurements, which provide records of changes in measured torque over time, have not been reported. In this study, a detailed torque measurement program, which was preverified by bone density measurements, was used at intervals of 0.05 s [11]. The recorded torque versus time waveforms allowed performing a systematic analysis. In addition, the accumulative torque values up to the maximum torque were observed to be larger for an incompatible/misfit superstructure than for a compatible/passive fit superstructure.

A clear misfit between the superstructure and implant body was confirmed by quantifying the tightening sensation, examining the radiographic images, and using a working cast model.

Since the distal screw tightening measurement was performed first, the accumulative torque on the mesial screw of the misfit superstructure was observed to be relatively large, with a relatively small torque slope up to the maximum torque. Notably, for the compatible/fit superstructure, the accumulative torques and time slopes of torques were almost the same for both the mesial and distal screws. The results of this study suggest that the method based on accumulative tightening torque is useful for evaluating the passive fit of the implant superstructure. In the future, a new method for evaluating passive fits can possibly be developed by examining the correlations between the waveforms and slopes of accumulative torques and the degree of misfit alignment between the superstructure and implant body. The limitation of this report is that the passive fit of the superstructure was evaluated by measuring the accumulated torque value; however, the evaluation was performed using different superstructure materials: resin-based and zirconia-based. The elastic modulus differs depending on the superstructure material, and the screw tightening torque behavior cannot be consistent. The findings are preliminary, that the comparison across materials is not methodologically robust, and that future studies should involve larger samples with consistent restorative materials.

## Figures and Tables

**Table 1:** Accumulative torques (expressed in units of Ncm) exerted on the tightening screws.

	**Mesial screw**	**Distal screw**
Misfit superstructure	511.3	390.4
Passive fit superstructure	262.3	219.5

## Data Availability

The data that support the findings of this study are available from the corresponding author upon reasonable request.
